# Association of Hoof Disorders with Inflammatory, Oxidative, Metabolic, and Milk Quality Biomarkers in Holstein–Friesian Dairy Cows

**DOI:** 10.3390/ijms27198610

**Published:** 2026-09-26

**Authors:** Jan Slósarz, Marek Balcerak, Małgorzata Kunowska-Slósarz, Grzegorz Grodkowski, Paweł Solarczyk, Piotr Kostusiak, Krzysztof Młynek, Kamila Puppel

**Affiliations:** 1 Institute of Animal Science, Warsaw University of Life Sciences, Ciszewskiego 8, 02-786 Warsaw, Poland; 2 Institute of Animal Science and Fisheries, University of Siedlce, ul. B. Prusa 14, 08-110 Siedlce, Poland

**Keywords:** dairy cows, hoof lesions, lameness, biomarkers, oxidative stress, inflammation, milk quality, acute-phase proteins, ROC analysis, principal component analysis

## Abstract

Hoof disorders are a major cause of reduced productivity and compromised welfare in dairy cows and are frequently accompanied by systemic inflammatory and metabolic disturbances. However, the relationships among different types of hoof lesions, oxidative stress, inflammation, metabolic status, and milk quality remain incompletely understood. Therefore, this study evaluated inflammatory, oxidative stress, metabolic, and milk quality biomarkers in Holstein–Friesian dairy cows with different hoof disorders and assessed their ability to discriminate lame from healthy animals. A total of 451 cows were classified as healthy (*n* = 210) or affected by horn (*n* = 190), infectious (*n* = 32), or degenerative (*n* = 13) hoof lesions; six cows with combinations of lesions that precluded unambiguous assignment to a single category were excluded from lesion-category comparisons. Blood and milk samples were analyzed for inflammatory cytokines, acute-phase proteins, oxidative stress markers, metabolic indicators, and milk quality traits. Compared with healthy cows, animals with hoof disorders, particularly those with infectious lesions, exhibited significantly higher concentrations of haptoglobin, serum amyloid A, IL-1β, IL-6, TNF-α, malondialdehyde, β-hydroxybutyrate, non-esterified fatty acids, cortisol, somatic cell count, and somatic cell score, whereas antioxidant biomarkers (total antioxidant capacity, glutathione peroxidase, superoxide dismutase, and catalase), IL-10, milk yield, fat content, and protein content were significantly lower (all *p* < 0.001). Correlation and principal component analyses revealed close associations among inflammatory, oxidative stress, metabolic, and milk quality variables. Receiver operating characteristic analysis identified IL-6 (AUC = 0.947), TNF-α (AUC = 0.934), haptoglobin (AUC = 0.921), and serum amyloid A (AUC = 0.913) as the biomarkers with the highest discriminatory performance for distinguishing clinically lame cows from healthy animals. Multivariate logistic regression identified β-hydroxybutyrate (OR = 3.24), malondialdehyde (OR = 2.26), somatic cell count (OR = 1.41), IL-6 (OR = 1.12), and TNF-α (OR = 1.09) as variables independently associated with lameness after evaluation of potential biological covariates. Overall, hoof disorders were associated with pronounced inflammatory, oxidative, and metabolic alterations accompanied by impaired milk quality. These findings suggest that IL-6, haptoglobin, serum amyloid A, and β-hydroxybutyrate may represent promising biomarkers for assessing and monitoring clinically established lameness in dairy cows. Further longitudinal studies are warranted to validate these findings and to evaluate the potential clinical utility of these biomarkers for lameness monitoring.

## 1. Introduction

Hoof disorders are among the most important health, welfare, and economic challenges in modern dairy production systems. Despite substantial advances in genetics, nutritional management, housing conditions, and preventive hoof care, lameness remains highly prevalent in dairy herds worldwide, affecting approximately 20–40% of cows depending on production system and herd management [1,2]. Beyond impairing locomotion, hoof disorders markedly reduce milk yield, impair reproductive performance, increase treatment and labor costs, and shorten productive lifespan, making them one of the leading causes of involuntary culling in high-producing dairy cows [3]. Consequently, improving hoof health has become a key objective of sustainable dairy farming and precision livestock management. Although these disorders originate within the claw, increasing evidence suggests that their biological consequences extend far beyond the locomotor system. Tissue injury and pain initiate coordinated systemic responses involving activation of inflammatory pathways, oxidative stress, endocrine regulation, and metabolic adaptation [4,5]. Therefore, hoof disorders are increasingly recognized as systemic diseases characterized by complex interactions among inflammation, oxidative stress, and metabolism rather than as isolated orthopedic conditions. The etiology of hoof disorders is multifactorial and reflects complex interactions among nutritional management, housing conditions, claw biomechanics, microbial pathogens, and host immunity [6].

Horn and infectious lesions account for a substantial proportion of lameness cases in dairy herds, although their prevalence varies among production systems [7,8]. Because the bovine claw is richly innervated, hoof disorders are considered among the most painful diseases affecting dairy cattle, resulting in prolonged activation of stress and inflammatory pathways [9]. Lameness is associated with reduced milk yield, impaired reproductive performance, and increased risk of premature culling, and may also be accompanied by alterations in milk composition and somatic cell count [10,11,12,13,14,15]. The development of hoof disorders reflects complex interactions among nutritional management, housing conditions, claw biomechanics, microbial pathogens, and host-related factors [16,17], while their clinical consequences commonly include pain, inflammation, impaired feed intake, and reduced productive performance [18,19]. These local pathological processes may be accompanied by systemic inflammatory, oxidative, and metabolic alterations [20].

Inflammation represents an important biological process associated with hoof disorders and systemic physiological alterations. Tissue injury activates innate immune responses characterized by increased production of pro-inflammatory cytokines, particularly interleukin-1β (IL-1β), interleukin-6 (IL-6), and tumor necrosis factor-α (TNF-α), which regulate leukocyte recruitment, endothelial activation, and hepatic synthesis of acute-phase proteins [21,22]. Among these mediators, IL-6 plays a pivotal role in integrating inflammatory and metabolic responses through regulation of acute-phase protein synthesis and energy metabolism [23]. In contrast, TNF-α and IL-1β amplify inflammatory signaling and contribute to tissue injury, whereas the anti-inflammatory cytokine IL-10 limits excessive immune activation and promotes restoration of tissue homeostasis. Consequently, the balance between pro- and anti-inflammatory mediators is considered a key determinant of disease progression and recovery [20,24].

The cytokine response is accompanied by increased hepatic synthesis of acute-phase proteins, particularly haptoglobin (Hp) and serum amyloid A (SAA), which are considered highly sensitive biomarkers of systemic inflammation in dairy cattle. Increased circulating concentrations of Hp and SAA have been consistently associated with infectious, inflammatory, and metabolic disorders and reflect the severity of tissue injury and immune activation [22,24]. Owing to their rapid response and high diagnostic sensitivity, these proteins have gained considerable attention as biomarkers for monitoring animal health [24]. Nevertheless, their combined relationship with oxidative stress, metabolic disturbances, and milk quality traits in cows affected by different hoof lesion types remains poorly understood.

Inflammatory activation is closely linked to oxidative stress, which develops when excessive production of reactive oxygen species (ROS) exceeds the capacity of antioxidant defense systems. Although ROS are essential mediators of host defense, their overproduction promotes lipid peroxidation, protein oxidation, mitochondrial dysfunction, and cellular damage [21]. Malondialdehyde (MDA) is one of the most widely used biomarkers of lipid peroxidation, whereas superoxide dismutase (SOD), glutathione peroxidase (GPx), catalase (CAT), and total antioxidant capacity (TAC) reflect the antioxidant status of the organism [25]. Disruption of redox homeostasis not only aggravates oxidative damage but also enhances inflammatory signaling through interactions between the NF-κB and Nrf2 pathways, thereby promoting disease progression [21]. Therefore, simultaneous evaluation of oxidative damage and antioxidant capacity may provide valuable insight into the systemic consequences of hoof disorders.

Metabolic adaptation is closely linked to inflammatory and oxidative responses in dairy cows. Reduced feed intake associated with pain and inflammation may contribute to negative energy balance, resulting in increased mobilization of non-esterified fatty acids (NEFA) and production of β-hydroxybutyrate (BHBA) [20,26,27]. These metabolic alterations may interact with inflammatory and redox processes, while activation of the hypothalamic–pituitary–adrenal axis and increased cortisol secretion may further contribute to changes in nutrient partitioning and immune regulation [23]. Such systemic alterations may also be accompanied by reduced milk yield and changes in milk quality [10,13,25]. Therefore, the combined assessment of inflammatory, oxidative stress, metabolic, and milk quality biomarkers may provide a more comprehensive characterization of hoof health status than individual biomarkers alone. Beyond serving as indicators of energy metabolism, these metabolites actively regulate immune cell activity, cytokine production, and redox homeostasis, highlighting the close interplay between metabolic and inflammatory pathways. Furthermore, activation of the hypothalamic–pituitary–adrenal axis results in increased cortisol secretion, contributing to metabolic adaptation, altered nutrient partitioning, and impaired immune regulation during chronic inflammatory conditions [23]. These immunometabolic alterations are frequently accompanied by reduced milk yield and impaired milk quality, reflected by increased somatic cell count (SCC) and somatic cell score (SCS), indicating that hoof disorders affect not only locomotor function but also mammary gland physiology and overall productive performance [10,13,25]. Therefore, the combined assessment of inflammatory, oxidative stress, metabolic, and milk quality biomarkers may provide a more comprehensive evaluation of hoof health than individual biomarkers alone.

Previous studies have predominantly focused on individual biomarkers or single disease entities, particularly mastitis, ketosis, and transition disorders, whereas comprehensive investigations integrating inflammatory cytokines, acute-phase proteins, oxidative stress biomarkers, metabolic indicators, and milk quality traits across different hoof lesion types are still limited [21,22]. Moreover, relatively few studies have combined these biomarkers with multivariate statistical approaches to simultaneously characterize biological interactions, evaluate discriminatory performance and identify variables independently associated with lameness. Integrating conventional biochemical analyses with correlation analysis, principal component analysis (PCA), receiver operating characteristic (ROC) analysis, and multivariate logistic regression may provide deeper insight into the systemic physiological alterations associated with hoof disorders and facilitate the identification of robust biomarker panels for herd health monitoring and precision livestock farming.

However, inflammatory, oxidative, metabolic, and stress-related biomarkers are inherently nonspecific and may be influenced by multiple physiological and pathological processes. Therefore, their alterations cannot be attributed exclusively to hoof disorders, and potential subclinical inflammatory or metabolic conditions may represent residual confounding factors. Given the cross-sectional design of the present study, these biomarkers were evaluated as indicators associated with clinically established hoof disorders rather than as predictors of future disease or markers of causal pathways.

We hypothesized that cows with clinically established hoof disorders would differ from clinically healthy cows in the concentrations and multivariate patterns of selected inflammatory, oxidative stress, metabolic, and milk quality biomarkers. Accordingly, the objective of this study was to evaluate these biomarkers in Holstein–Friesian dairy cows affected by different hoof lesion types and to characterize their associations with clinically established lameness. By integrating conventional laboratory measurements with multivariate statistical analyses, including PCA, ROC analysis, and multivariate logistic regression, we aimed to identify biomarker patterns associated with hoof health status and to assess their discriminatory value for distinguishing clinically lame from healthy cows under commercial dairy farming conditions.

## 2. Results

The concentrations of inflammatory, oxidative stress, and metabolic biomarkers differed significantly among the evaluated hoof lesion groups (Table 1). Cows with infectious hoof lesions exhibited the highest concentrations of haptoglobin, serum amyloid A, IL-1β, IL-6, and TNF-α, whereas healthy cows consistently showed the lowest values (all *p* < 0.001). In contrast, IL-10 displayed the opposite pattern, with the highest concentration observed in healthy cows and the lowest in cows affected by infectious lesions (*p* < 0.001). Markers of oxidative stress also varied significantly according to hoof health status. MDA concentrations progressively increased from healthy cows to those with infectious lesions, while antioxidant biomarkers (TAC, GPx, SOD, and catalase) showed a gradual decline, reaching their lowest values in cows with infectious hoof lesions (all *p* < 0.001). Similarly, metabolic biomarkers were markedly affected by hoof disorders. Concentrations of BHBA, NEFA, and cortisol increased significantly in lame cows, with the highest values recorded in animals with infectious lesions (all *p* < 0.001). Overall, cows with horn lesions generally exhibited intermediate biomarker concentrations between healthy animals and those with infectious lesions, whereas degenerative lesions were characterized by a less pronounced metabolic and inflammatory response.

Significant correlations were observed between milk quality traits and inflammatory biomarkers (Table 2). Somatic cell count and somatic cell score were strongly positively correlated (*r* = 0.94, *p* < 0.001) and were positively associated with IL-6, TNF-α, serum amyloid A, and malondialdehyde (all *p* < 0.001). Among the inflammatory biomarkers, IL-6 showed the strongest positive correlation with TNF-α (*r* = 0.81), followed by SAA (*r* = 0.76) and MDA (*r* = 0.69). Similarly, TNF-α was positively correlated with SAA (*r* = 0.72) and MDA (*r* = 0.67), while SAA was also strongly associated with MDA (*r* = 0.71) (all *p* < 0.001). Milk yield was negatively correlated with all evaluated inflammatory and oxidative stress biomarkers, with the strongest inverse association observed for IL-6 (*r* = −0.52), followed by TNF-α (*r* = −0.49), SCC (*r* = −0.48), SCS (*r* = −0.45), SAA (*r* = −0.44), and MDA (*r* = −0.39) (all *p* < 0.001). Overall, these findings indicate that increasing inflammatory activity and oxidative stress were associated with poorer milk quality and reduced milk production.

Principal component analysis (PCA) revealed distinct clustering of milk quality, inflammatory, oxidative stress, and metabolic biomarkers (Table 3). The first principal component (PC1) was characterized by strong positive loadings for inflammatory markers (IL-6, TNF-α, haptoglobin, serum amyloid A, and IL-1β), oxidative stress markers MDA, SCC, and SCS, and metabolic indicators (BHBA, NEFA, and cortisol). In contrast, milk yield, total antioxidant capacity, and glutathione peroxidase showed strong negative loadings on PC1, indicating an inverse association between inflammatory and metabolic disturbances and milk production as well as antioxidant status. The second principal component (PC2) was mainly associated with milk composition, with fat and protein exhibiting the highest positive loadings. Communality values ranged from 0.499 to 0.850, with the highest values observed for IL-6, TNF-α, serum amyloid A, haptoglobin, MDA, and SCC, indicating that these variables contributed most strongly to the overall multivariate structure.

The first two principal components explained 71.2% of the total variance (PC1 = 55.4%, PC2 = 15.8%), indicating that these components adequately summarized the relationships among the evaluated biomarkers.

Receiver operating characteristic (ROC) analysis demonstrated that all evaluated biomarkers significantly discriminated clinically lame cows from healthy animals (Table 4). Among the evaluated biomarkers, IL-6 showed the highest discriminatory performance, followed by TNF-α, haptoglobin, and serum amyloid A, all of which exhibited excellent discrimination (AUC > 0.90). The oxidative stress marker malondialdehyde also demonstrated excellent discriminatory performance (AUC = 0.902), whereas somatic cell count and β-hydroxybutyrate showed good discriminatory ability, with AUC values of 0.887 and 0.861, respectively. IL-6 achieved the highest sensitivity (91.3%), specificity (88.6%), and Youden index (0.799), indicating the greatest ability to distinguish clinically lame cows from healthy animals within the study population. All evaluated biomarkers significantly discriminated lame cows from healthy animals (*p* < 0.001).

Multivariate logistic regression identified all evaluated biomarkers as variables independently associated with lameness (Table 5). IL-6 and TNF-α remained significantly associated with lameness, with IL-6 showing the strongest contribution to the model. Among all evaluated variables, β-hydroxybutyrate exhibited the highest odds ratio (OR = 3.24), followed by malondialdehyde (OR = 2.26) and somatic cell count (OR = 1.41), indicating that metabolic imbalance, oxidative stress, and mammary gland health were independently associated with lameness. All variables remained highly significant (*p* < 0.001), while the Wald χ^2^ statistics confirmed IL-6 as the most influential variable in the multivariate model.

Receiver operating characteristic (ROC) curve analysis confirmed the high discriminatory performance of the evaluated biomarkers for discriminating lame cows from healthy animals (Figure 1). Among the evaluated variables, IL-6 showed the highest discriminatory ability, followed by TNF-α, haptoglobin, serum amyloid A, malondialdehyde, somatic cell count, and β-hydroxybutyrate. All ROC curves were located well above the reference line, indicating good to excellent discriminatory ability of the evaluated biomarkers. Inflammatory biomarkers demonstrated the greatest discriminatory performance, whereas oxidative stress, metabolic, and milk quality indicators showed slightly lower, although still high, discriminatory performance.

Principal component analysis (PCA) revealed a clear separation between healthy cows and animals affected by hoof disorders based on inflammatory, oxidative stress, metabolic, and milk production variables (Figure 2). Healthy cows clustered on the negative side of PC1 and were primarily associated with higher milk yield, total antioxidant capacity, glutathione peroxidase, and superoxide dismutase. In contrast, cows with hoof lesions were distributed on the positive side of PC1 and were closely associated with increased concentrations of inflammatory (IL-6, TNF-α, haptoglobin, and serum amyloid A), oxidative stress (MDA), metabolic (BHBA, NEFA, and cortisol), and milk quality (SCC and SCS) biomarkers. Among the affected groups, cows with infectious lesions showed the greatest separation from healthy animals, whereas horn and degenerative lesions occupied intermediate positions. Overall, the PCA biplot highlighted a clear association between hoof disorders and systemic inflammation, oxidative stress, and metabolic imbalance, while healthy cows were characterized by greater antioxidant capacity and higher milk production.

The multivariate logistic regression model identified all evaluated biomarkers as variables independently associated with lameness (Figure 3). Among the analyzed variables, β-hydroxybutyrate showed the strongest association with lameness, followed by MDA and somatic cell count. IL-6 and TNF-α also remained independently associated with lameness, although their effect sizes were smaller. All odds ratios were greater than 1, and the corresponding 95% confidence intervals did not cross the null value, indicating significant positive associations between the evaluated biomarkers and lameness. Overall, the multivariate model demonstrated that metabolic, oxidative, inflammatory, and milk-quality biomarkers were independently associated with clinically established lameness.

## 3. Discussion

The inflammatory response differed clearly among hoof lesion types. Cows with infectious lesions showed the highest concentrations of IL-1β, IL-6, TNF-α, haptoglobin, and serum amyloid A, whereas healthy cows had the lowest values. The high inflammatory response observed in infectious lesions is biologically plausible. Digital dermatitis, interdigital dermatitis, and interdigital phlegmon involve bacterial infection and tissue destruction, which may continuously stimulate innate immune mechanisms [6,28]. Under such conditions, cytokines such as IL-1β, IL-6, and TNF-α promote leukocyte recruitment, endothelial activation, and hepatic acute-phase protein synthesis. This was reflected in the present study by the marked increase in haptoglobin and serum amyloid A, which reached their highest concentrations in cows with infectious lesions. Similar systemic increases in acute-phase proteins have also been reported in dairy cows affected by sole ulcer, white line disease, and digital dermatitis, supporting an association between hoof lesions and measurable systemic inflammatory alterations [28]. Similar links between cytokine activation, acute-phase response, and metabolic disruption have been reported in dairy cows with inflammatory and metabolic disorders [5,20,23,28]. Among the cytokines evaluated, IL-6 appears to be particularly relevant. The central role of IL-6 in hoof disorders has also been highlighted by Pirkkalainen et al. [29], who demonstrated that digital dermatitis was accompanied by both local and systemic inflammatory activation characterized by increased IL-6 concentrations. IL-6 showed the highest discriminatory performance in the ROC analysis and the strongest contribution to the logistic regression model, suggesting that IL-6 may be one of the most informative inflammatory biomarkers of lameness in this dataset. This is consistent with the biological role of IL-6 as a mediator connecting inflammation, hepatic acute-phase protein synthesis, and energy metabolism [23,24]. In contrast, the reduction in IL-10 may indicate insufficient anti-inflammatory compensation in lame cows, especially in those with infectious lesions. The increase in pro-inflammatory cytokines was accompanied by a marked elevation in circulating haptoglobin and serum amyloid A concentrations, confirming activation of the acute-phase response. Both proteins reached their highest values in cows with infectious hoof lesions, supporting the close relationship between local tissue infection and systemic inflammatory activation. Haptoglobin and serum amyloid A are synthesized primarily by the liver following stimulation with IL-6 and other pro-inflammatory cytokines and are considered among the most sensitive biomarkers of inflammation in cattle [24]. Similar increases have been described in cows affected by mastitis, metritis, respiratory diseases, and transition disorders, where circulating concentrations of acute-phase proteins closely reflect disease severity and tissue injury [24]. Our results are consistent with similar systemic inflammatory alterations in cows with hoof disorders, particularly infectious lesions, suggesting that both proteins may be useful indicators of systemic inflammation in clinically lame cows. From a practical standpoint, combining measurements of inflammatory cytokines with acute-phase proteins may provide a more comprehensive characterization of systemic inflammatory status in clinically lame cows.

Evidence of oxidative stress was also apparent in the present study. Cows with hoof disorders, particularly those affected by infectious lesions, showed higher plasma MDA concentrations together with lower total antioxidant capacity and reduced activities of antioxidant enzymes. These findings indicate that inflammatory activation was accompanied by increased oxidative damage and reduced antioxidant defense. Although ROS play an essential role in antimicrobial defense and intracellular signaling, their sustained production promotes lipid peroxidation, protein oxidation, mitochondrial dysfunction, and delayed tissue repair [5,21]. Because MDA is generated during oxidation of membrane polyunsaturated fatty acids, its accumulation reflects excessive production of reactive oxygen species and disruption of cellular redox balance. Similar increases in MDA have been reported in dairy cows affected by inflammatory and metabolic disorders, including ketosis, mastitis, and transition-related diseases, supporting the close relationship between oxidative stress and systemic inflammation [20]. These observations agree with Fritzen et al. [30], who emphasized that oxidative stress is a common feature of inflammatory diseases in dairy cattle and contributes to impaired immune function and tissue repair. The higher MDA concentrations observed in cows with infectious lesions are consistent with the close relationship between inflammatory activation and oxidative stress described in dairy cattle [20,21]. The oxidative imbalance identified in the present study was further supported by reduced total antioxidant capacity and lower activities of SOD, GPx, and CAT. These enzymes constitute the first line of antioxidant defense by converting superoxide radicals and hydrogen peroxide into less reactive molecules, thereby protecting lipids, proteins, and nucleic acids against oxidative injury. Their reduced activity is consistent with lower antioxidant defense in cows with clinically established hoof disorders. Similar reductions in enzymatic antioxidant activity have been described in cows experiencing metabolic stress and inflammatory diseases, where impaired antioxidant defense has been associated with reduced immune competence and increased susceptibility to tissue damage [21,25].

The simultaneous increase in oxidative damage and decline in antioxidant protection indicates that oxidative stress is closely integrated with the inflammatory response accompanying hoof disorders. This interaction is biologically plausible because excessive ROS production not only damages cellular structures but also activates redox-sensitive signaling pathways, including NF-κB, thereby enhancing the expression of pro-inflammatory cytokines and perpetuating the inflammatory response [5,21]. These reciprocal interactions between oxidative stress and inflammatory signaling have been described in dairy cattle and provide biological context for the concurrent alterations observed in the present study [5,21]. The combined evaluation of MDA and antioxidant biomarkers may provide a more comprehensive characterization of oxidative alterations associated with clinically established hoof disorders than assessment of a single oxidative stress indicator alone. The metabolic profile of lame cows indicates that clinically established hoof disorders were associated with substantial alterations in energy metabolism. In the present study, cows with infectious and degenerative lesions exhibited higher circulating concentrations of NEFA and BHBA and increased cortisol levels, suggesting consistency with alterations in energy balance. The direction of this association, however, cannot be established from the present cross-sectional data. Pain, inflammation, and impaired locomotion associated with hoof disorders may reduce feed intake and promote negative energy balance, adipose tissue mobilization, and hepatic ketogenesis. Conversely, pre-existing negative energy balance and increased lipid mobilization may alter immune and metabolic homeostasis and potentially contribute to susceptibility to claw horn lesions. Thus, the elevated BHBA and NEFA concentrations observed in lame cows should be interpreted as metabolic alterations associated with hoof health status rather than as evidence of a unidirectional causal effect of hoof disorders on energy metabolism [4,27]. Similar metabolic adaptations have been described in dairy cows during periods of inflammatory stress, supporting the close relationship between energy metabolism and immune activation [4,31]. Among the evaluated metabolites, BHBA appears to be of particular interest because its biological significance extends beyond its role as a marker of negative energy balance. The close interaction between lipid mobilization, inflammatory signaling, and immune dysfunction has also been emphasized in recent reviews describing immunometabolic adaptations during disease in dairy cows [31]. Experimental and clinical studies have shown that elevated BHBA concentrations impair neutrophil chemotaxis, phagocytic activity, and oxidative burst, while simultaneously promoting oxidative stress and inflammatory signaling [4,20]. Likewise, increased NEFA concentrations are not only indicative of enhanced lipid mobilization but may also modulate inflammatory signaling and immune function [4,31]. Therefore, the simultaneous elevation of BHBA and NEFA in lame cows may reflect the close interaction between metabolic and inflammatory status rather than isolated changes in energy metabolism [4,31]. The coexistence of elevated cortisol with increased inflammatory cytokines and oxidative stress biomarkers observed in the present study suggests a close relationship among endocrine, metabolic, inflammatory, and oxidative responses in cows with clinically established hoof disorders [5,31]. An additional feature of the metabolic response was the increase in circulating cortisol concentrations. Because hoof disorders are associated with persistent pain and reduced mobility, activation of the hypothalamic–pituitary–adrenal axis is an expected physiological response. Cortisol supports short-term metabolic adaptation by promoting gluconeogenesis and lipid mobilization; however, prolonged elevation may impair immune function, delay tissue repair, and increase susceptibility to secondary infections [5]. The coexistence of elevated cortisol with increased inflammatory cytokines and oxidative stress biomarkers observed in the present study suggests a close relationship among endocrine, metabolic, inflammatory, and oxidative responses in cows with clinically established hoof disorders [5,31]. Taken together, these observations indicate that metabolic disturbances associated with hoof lesions should be interpreted within the broader context of immunometabolic adaptation. Rather than representing isolated changes in energy metabolism, alterations in NEFA, BHBA, and cortisol should be interpreted as components of the broader systemic biomarker profile associated with clinically established hoof disorders. Their temporal and causal relationships with hoof lesion development cannot be determined from the present cross-sectional study. The significant correlations observed among inflammatory cytokines, acute-phase proteins, oxidative stress biomarkers, and metabolic indicators indicate that these physiological processes are closely interconnected in cows with clinically established hoof disorders. Rather than representing independent physiological alterations, inflammation, oxidative stress, and disturbed energy metabolism appear to constitute different components of the systemic alterations associated with hoof disorders [5,31]. This observation is consistent with the concept of immunometabolism, which emphasizes the reciprocal interactions between immune activation and metabolic regulation during disease [31]. Therefore, simultaneous evaluation of NEFA, BHBA, cortisol, and inflammatory biomarkers may provide a more comprehensive assessment of the systemic consequences of hoof disorders than interpretation of individual metabolic variables alone.

The metabolic and inflammatory disturbances identified in the present study were accompanied by reduced milk production and deterioration of udder health. Lame cows produced less milk and had significantly higher SCC and SCS values than healthy animals, confirming that the biological consequences of hoof disorders extend beyond impaired locomotion. Similar reductions in milk yield have been consistently reported in lame dairy cows and are generally attributed to lower dry matter intake, reduced feeding activity, and redistribution of nutrients toward maintenance of immune function instead of milk synthesis [10,13,14]. Our findings support these observations and further indicate that systemic inflammatory alterations were associated with reduced productive performance. Higher SCC values were also observed in lame cows. Although cows with clinical mastitis were excluded, the presence of subclinical intramammary infection cannot be completely ruled out; therefore, the higher SCC observed in cows with hoof disorders cannot be attributed specifically to systemic inflammation. Nevertheless, the observed associations between SCC and inflammatory biomarkers suggest a relationship between mammary gland health indicators and systemic inflammatory status that warrants further investigation [32,33]. Similar relationships between systemic inflammation, increased SCC, and impaired milk quality have been described in dairy cows affected by inflammatory and metabolic diseases [25,33]. Interestingly, the changes observed in milk composition were less pronounced than those detected for inflammatory and metabolic biomarkers. Identification of biomarker panels with high discriminatory performance may provide a more objective assessment of the systemic response associated with clinically established hoof disorders. Such biomarker profiles may complement clinical examination and locomotion scoring and support monitoring and clinical decision-making; however, the present data do not establish their ability to predict a specific hoof disorder before its clinical manifestation. An important strength of the present study is the combined use of conventional laboratory measurements and multivariate statistical analyses. Evaluation of individual biomarkers provides information on selected physiological processes; however, it does not fully reflect the complex biological interactions accompanying hoof disorders. By combining correlation analysis, principal component analysis, receiver operating characteristic analysis, and multivariate logistic regression, it was possible to evaluate the relationships among inflammatory, oxidative stress, metabolic, and milk quality biomarkers simultaneously, providing a broader picture of the systemic response associated with different hoof lesion types.

The discriminatory value of the evaluated biomarkers was supported by ROC analysis and multivariate logistic regression. Among all variables, IL-6, haptoglobin, serum amyloid A, MDA, BHBA, and SCC showed the highest ability to discriminate lame cows from healthy animals. Moreover, logistic regression demonstrated that these biomarkers remained significantly associated with lameness after adjustment for other variables included in the model. These observations support the potential value of combining biomarkers representing different physiological processes to characterize the systemic alterations associated with lameness. This conclusion is consistent with the recent scoping review by Sadiq et al. [34], which emphasized that no single biomarker is sufficiently sensitive or specific for lameness diagnosis and recommended the development of integrated biomarker panels combining inflammatory, oxidative stress, metabolic, and production-related indicators. To our knowledge, relatively few studies have applied such an integrated analytical approach to evaluate systemic biomarker profiles in dairy cows affected by different hoof lesion types, highlighting the novelty of the present work. From a practical perspective, multimarker assessment may complement clinical examination and locomotion scoring by providing objective information on the systemic physiological status of cows with clinically established lameness.

The present study has several limitations that should be considered when interpreting the results. Biomarker concentrations were determined at a single sampling point; therefore, temporal changes occurring during disease progression and recovery could not be evaluated. In addition, although all animals originated from the same commercial herd and were managed under uniform conditions, the influence of individual environmental or management-related factors cannot be completely excluded. Future longitudinal studies including repeated sampling before and after treatment would provide valuable information on biomarker dynamics and help determine their usefulness for monitoring disease progression and therapeutic responses. Although cows with clinically apparent concurrent inflammatory or metabolic diseases were excluded following veterinary examination, the possible presence of subclinical conditions cannot be completely ruled out. In addition, because biomarker concentrations were determined at a single sampling point, the temporal sequence between biomarker alterations and hoof lesion development cannot be established. Consequently, the present cross-sectional design does not allow determination of whether the observed inflammatory, oxidative, and metabolic alterations preceded lesion development or occurred as a consequence of established disease. Therefore, the discriminatory performance observed in the present study should be interpreted as the ability to distinguish clinically lame from healthy cows at the time of examination and not as evidence of prospective prediction of specific hoof disorders before their clinical manifestation. Despite these limitations, the study offers a comprehensive assessment of inflammatory, oxidative stress, metabolic, and milk quality biomarkers in dairy cows with different categories of hoof lesions. Integrating conventional biochemical measurements with PCA, ROC analysis, and multivariate logistic regression provided new insight into the systemic nature of hoof disorders and identified several biomarkers associated with clinically established lameness. Collectively, the present findings complement recent evidence indicating that objective laboratory biomarkers may become an important component of precision livestock farming strategies for monitoring hoof health in dairy cattle [34].

## 4. Materials and Methods

### 4.1. Animals, Housing, and Experimental Design

The study was carried out on 451 Polish Holstein–Friesian dairy cows maintained under commercial production conditions in a free-stall dairy herd. Prior to inclusion in the study, all cows underwent a general clinical health examination performed by a veterinarian. Animals showing clinical signs of concurrent diseases or disorders that could affect systemic inflammatory or metabolic status, including clinical mastitis, metritis, ketosis, or other clinically apparent inflammatory or metabolic conditions, were excluded from the study. All animals underwent a comprehensive clinical hoof examination during routine functional hoof trimming performed by the same experienced professional hoof trimmer. After thorough cleaning, each claw was carefully examined, and hoof lesions were diagnosed and classified according to the ICAR Claw Health Atlas (International Committee for Animal Recording, ICAR) into horn, infectious, and degenerative lesions. Only cows presenting clinically relevant hoof lesions were assigned to the lame group, whereas cows without detectable hoof lesions served as healthy controls. Based on the clinical examination, cows were classified as healthy or lame (Table 6).

The general characteristics of the experimental groups are presented in Table 6, while milk production and milk quality traits are summarized in Table 7. Compared with healthy cows, lame animals produced less milk (8912 vs. 9845 kg/year; *p* < 0.001) and exhibited significantly lower milk fat and protein contents. In contrast, hoof disorders were associated with markedly higher somatic cell count (391 vs. 182 × 10^3^ cells/mL, *p* < 0.001) and somatic cell score (4.08 vs. 2.63, *p* < 0.001), whereas the fat-to-protein ratio did not differ between groups (*p* = 0.881).

The same trained hoof trimmer performed all hoof examinations and lesion classifications to ensure consistency of the diagnostic procedure. Lame cows were categorized according to the type of hoof lesion into horn lesions (*n* = 190), infectious lesions (*n* = 32), and degenerative lesions (*n* = 13). Table 8 additionally summarizes the distribution of individual hoof diagnoses recorded during clinical examination; therefore, the number of individual lesion diagnoses may exceed the number of cows because more than one lesion could be recorded in the same animal. Horn lesions represented the predominant category, accounting for 78.8% of all hoof disorders, whereas infectious and degenerative lesions accounted for 13.3% and 5.4%, respectively (Table 8). Among individual lesions, sole ulcer was the most prevalent disorder (32.4%), followed by double sole (16.2%), sole hemorrhage (13.7%), and digital dermatitis (10.8%). Because lesion classification was performed during routine clinical hoof examination, blinding of the examiner was not feasible.

Following clinical examination and group allocation, blood and milk samples were collected from all animals before the initiation of any therapeutic intervention, including the administration of non-steroidal anti-inflammatory drugs (NSAIDs), analgesics, or antimicrobial treatment. This sampling protocol minimized the potential influence of treatment on inflammatory, oxidative stress, metabolic, and milk quality biomarkers. Blood samples were used for the determination of inflammatory, oxidative stress, and metabolic biomarkers, whereas milk samples were analyzed for milk composition, somatic cell count, and additional milk quality indicators.

The study was observational and cross-sectional and was not designed to experimentally test causal mechanisms or specific inflammatory, oxidative, or metabolic pathways. The measured biomarkers were used to characterize systemic physiological differences associated with clinically established hoof disorders.

### 4.2. Determination of Milk Production and Milk Quality Traits

Milk yield data were obtained from the herd management recording system and corresponded to the official test-day milk recording performed on the day of blood and milk sampling. Individual milk yield (kg/day) was automatically recorded during milking using the on-farm milking management system.

Milk samples were collected during the morning milking and analyzed for fat, protein, lactose, casein, and milk density using a MilkoScan FT120 analyzer (FOSS Electric A/S, Hillerød, Denmark). Somatic cell count was determined using a Somacount 150 analyzer (Bentley Instruments Inc., Chaska, MN, USA). The somatic cell score was calculated according to the equation: SCS = log2 (SCC/100) + 3, where SCC is expressed as ×10^3^ cells/mL. Calibration of both analyzers was performed using certified reference milk standards supplied by the National Animal Breeding Centre (Warsaw, Poland) according to the manufacturers’ recommendations. The fat-to-protein ratio (FPR) was calculated from the measured fat and protein concentrations.

### 4.3. Determination of Metabolic Biomarkers

Blood samples were collected from the coccygeal vein into serum tubes (Vacutainer^®^, Becton Dickinson, Franklin Lakes, NJ, USA) immediately after the clinical examination and before morning feeding. Samples were centrifuged at 3000× *g* for 15 min, and the obtained serum was stored at −20 °C until analysis. Serum concentrations of glucose, non-esterified fatty acids, β-hydroxybutyrate, aspartate aminotransferase, and gamma-glutamyl transferase were determined using an automatic biochemical analyzer (BS-800M; PZ Cormay S.A., Warsaw, Poland) according to the manufacturer’s instructions. Commercial veterinary diagnostic kits (PZ Cormay S.A., Warsaw, Poland) were used for the determination of glucose, AST, and GGT activities, whereas NEFA and BHBA concentrations were measured using enzymatic colorimetric assays routinely applied for the evaluation of energy balance in dairy cattle. All analyses were performed in duplicate under standardized laboratory conditions, and internal quality control procedures were implemented throughout the analytical period. The measured metabolites were used to assess the energy status and metabolic adaptation of dairy cows in relation to hoof health status.

### 4.4. Determination of Oxidative Stress Biomarkers

Oxidative stress was evaluated by determining plasma concentrations of malondialdehyde and the activities of catalase, glutathione peroxidase, superoxide dismutase, and total antioxidant capacity.

Plasma MDA concentration was determined using the thiobarbituric acid reactive substances (TBARS) assay. Briefly, plasma samples were mixed with trichloroacetic acid and thiobarbituric acid, incubated at 90 °C for 45 min, cooled, centrifuged, and the absorbance of the reaction product was measured at 532 nm using a NanoQuant Infinite M200 Pro microplate reader (Tecan Austria GmbH, Grödig, Austria).

Activities of CAT, GPx, and SOD, together with TAC, were determined spectrophotometrically using commercially available assay kits according to the manufacturers’ instructions. GPx activity was measured using the Ransel kit (Cat. No. RS505), SOD activity using the Ransod kit (Cat. No. SD126), TAC using the Total Antioxidant Status kit (Cat. No. NX2331) (Randox Laboratories Ltd., Crumlin, UK), and catalase activity using the Catalase Assay Kit (Cat. No. 707002; Cayman Chemical, Ann Arbor, MI, USA). All samples were analyzed in duplicate, and internal quality control procedures were applied throughout the analytical period to ensure analytical precision and reproducibility. The Ransel assay exhibited a within-run precision of <4.86% according to the manufacturer’s specifications.

### 4.5. Determination of Inflammatory and Mammary Gland Biomarkers

Blood samples were collected from the coccygeal vein into serum tubes (Vacutainer^®^, Becton Dickinson, Franklin Lakes, NJ, USA), centrifuged at 3000× *g* for 15 min, and serum samples were stored at −20 °C until analysis. Serum concentrations of interleukin-1β (IL-1β), interleukin-6 (IL-6), tumor necrosis factor-α (TNF-α), interleukin-10 (IL-10), haptoglobin (Hp), and serum amyloid A (SAA) were determined using commercially available bovine enzyme-linked immunosorbent assay (ELISA) kits (MyBioSource Inc., San Diego, CA, USA) according to the manufacturer’s instructions. Optical density was measured using a NanoQuant Infinite M200 Pro microplate reader (Tecan Austria GmbH, Grödig, Austria), and biomarker concentrations were calculated from standard calibration curves.

All analyses were performed in duplicate under standardized laboratory conditions, and internal quality control procedures were applied throughout the analytical period.

### 4.6. Statistical Analysis

Statistical analyses were performed using Statistica 13.3 (TIBCO Software Inc., Palo Alto, CA, USA). Data are presented as mean ± standard deviation (SD). The normality of data distribution was assessed using the Shapiro–Wilk test, whereas homogeneity of variances was evaluated using Levene’s test. Variables fulfilling the assumptions of normality and homogeneity were analyzed using parametric statistical methods. Because the number of animals differed among the evaluated hoof lesion categories, these assumptions were verified before applying one-way analysis of variance (ANOVA) to ensure the validity of the parametric analyses in the presence of unequal group sizes.

Differences among healthy cows and cows with horn, infectious, and degenerative hoof lesions were evaluated using one-way ANOVA followed by Tukey’s honestly significant difference (HSD) post hoc test. Comparisons between healthy and lame cows were performed using the independent-samples Student’s *t*-test. The magnitude of differences between groups was assessed using Cohen’s *d* effect size and interpreted as small (*d* = 0.20), medium (*d* = 0.50), or large (*d* ≥ 0.80).

Relationships among inflammatory, oxidative stress, metabolic, and milk quality variables were assessed using Pearson’s product–moment correlation analysis. Correlation coefficients were interpreted as weak (*r* < 0.30), moderate (0.30 ≤ *r* < 0.70), or strong (*r* ≥ 0.70).

To explore multivariate relationships among the evaluated variables, principal component analysis (PCA) was performed using standardized data based on the correlation matrix. Principal components with eigenvalues greater than 1.0 were retained according to the Kaiser criterion. Variable loadings and communality values were calculated to determine the contribution of individual variables to the extracted components.

The discriminatory performance of selected biomarkers for discriminating lame cows from healthy animals at the time of clinical examination was evaluated using receiver operating characteristic (ROC) curve analysis. The area under the ROC curve (AUC), 95% confidence intervals (95% CI), optimal cut-off values, sensitivity, specificity, and the Youden index were calculated for each biomarker. ROC-derived cut-off values were interpreted as study-specific discriminatory thresholds and not as validated prospective diagnostic or predictive cut-offs.

Multivariate logistic regression analysis was performed to evaluate the independent associations between selected biomarkers and lameness. Potential biological covariates, including days in milk (DIM), parity, age, body condition score (BCS), milk yield, season, and stage of lactation, were initially evaluated during model development. As none of these variables showed a significant association with lameness (*p* > 0.05), they were not retained in the final model. Nevertheless, residual confounding by unmeasured factors or undetected subclinical conditions cannot be excluded. Biomarkers were subsequently entered into the multivariate logistic regression model using a stepwise selection procedure to identify variables independently associated with lameness while minimizing redundancy among correlated biomarkers. The final multivariate model included biomarkers that remained significantly associated with lameness after evaluation of the potential biological covariates. Regression coefficients (β), standard errors (SE), odds ratios (ORs) with 95% confidence intervals (95% CI), and Wald χ^2^ statistics were calculated for each variable. Given the cross-sectional design, odds ratios were interpreted as measures of association with clinically established lameness at the time of examination and not as estimates of prospective disease risk or causal effects. The statistical models were not intended to establish temporal or mechanistic relationships among the evaluated biomarkers and hoof disorders.

A two-sided *p*-value < 0.05 was considered statistically significant.

## 5. Conclusions

The present study provides comprehensive evidence that hoof disorders are associated with pronounced systemic inflammatory, oxidative stress, and metabolic alterations accompanied by impaired milk production and milk quality in dairy cows. Among the evaluated lesion types, infectious hoof lesions were associated with the most pronounced changes in inflammatory cytokines, acute-phase proteins, oxidative stress biomarkers, and metabolic indicators, highlighting their close relationship with systemic physiological disturbances. Correlation analysis and principal component analysis revealed close relationships among these biological processes, whereas ROC analysis and multivariate logistic regression identified IL-6, TNF-α, haptoglobin, serum amyloid A, malondialdehyde, β-hydroxybutyrate, and somatic cell count as biomarkers strongly associated with lameness and exhibiting high discriminatory performance. The findings indicate that no single biomarker fully reflects the complex biological alterations accompanying hoof disorders. Rather, the combined evaluation of inflammatory, oxidative stress, metabolic, and milk quality biomarkers may provide a more comprehensive assessment of the systemic changes associated with lameness than individual biomarkers alone. Such an integrated approach may complement clinical examination and support herd health monitoring within precision livestock farming systems. Overall, this study expands current knowledge of the systemic biological alterations associated with hoof disorders and identifies several biomarkers with potential utility for lameness assessment under commercial dairy farming conditions. Future longitudinal and multicenter studies are warranted to validate these findings across different breeds, production systems, and stages of lactation and to determine the clinical applicability of these biomarkers for lameness monitoring.

## Figures and Tables

**Figure 1:**
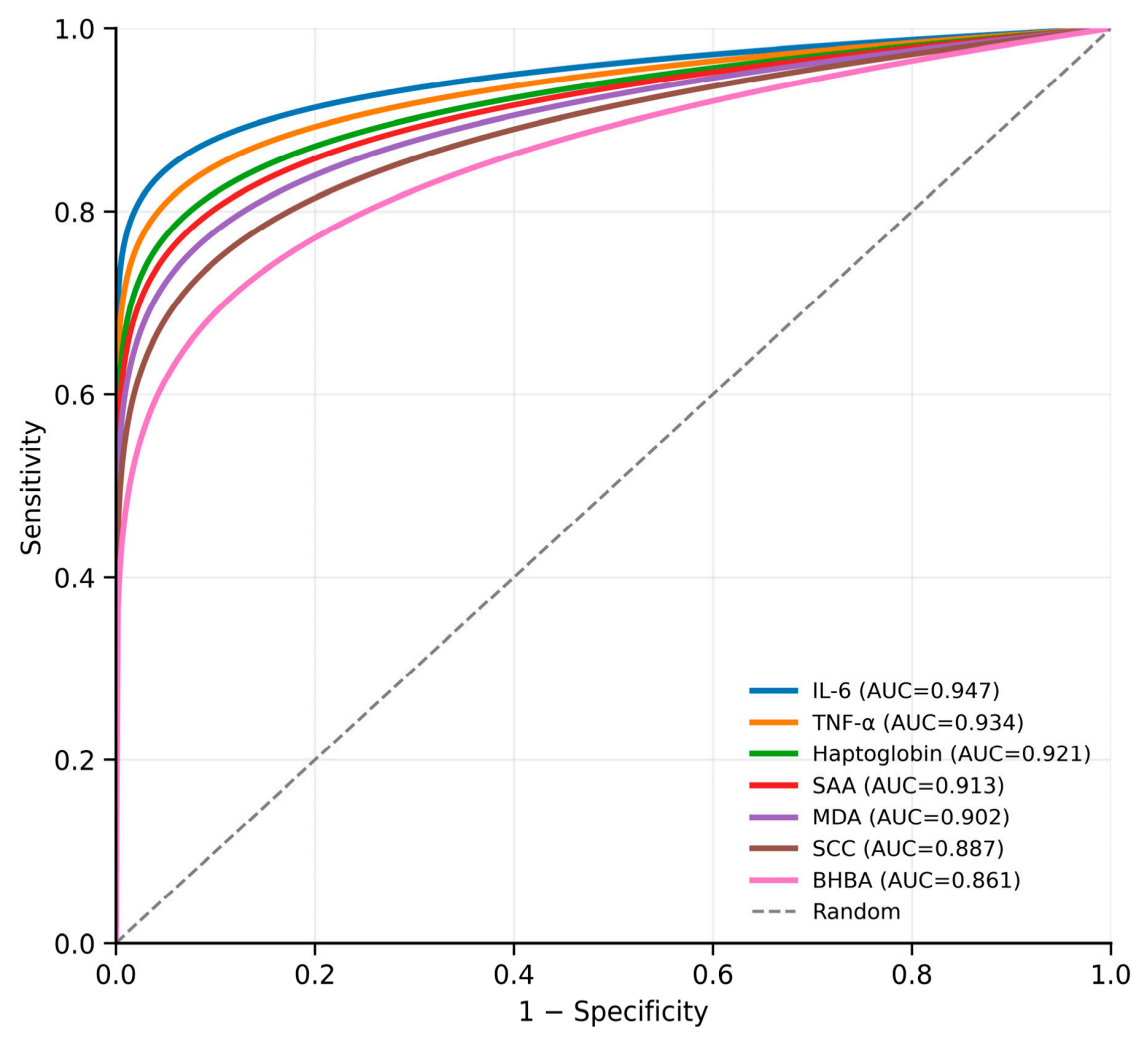
Receiver operating characteristic (ROC) curves for selected biomarkers discriminating lame cows.

**Figure 2:**
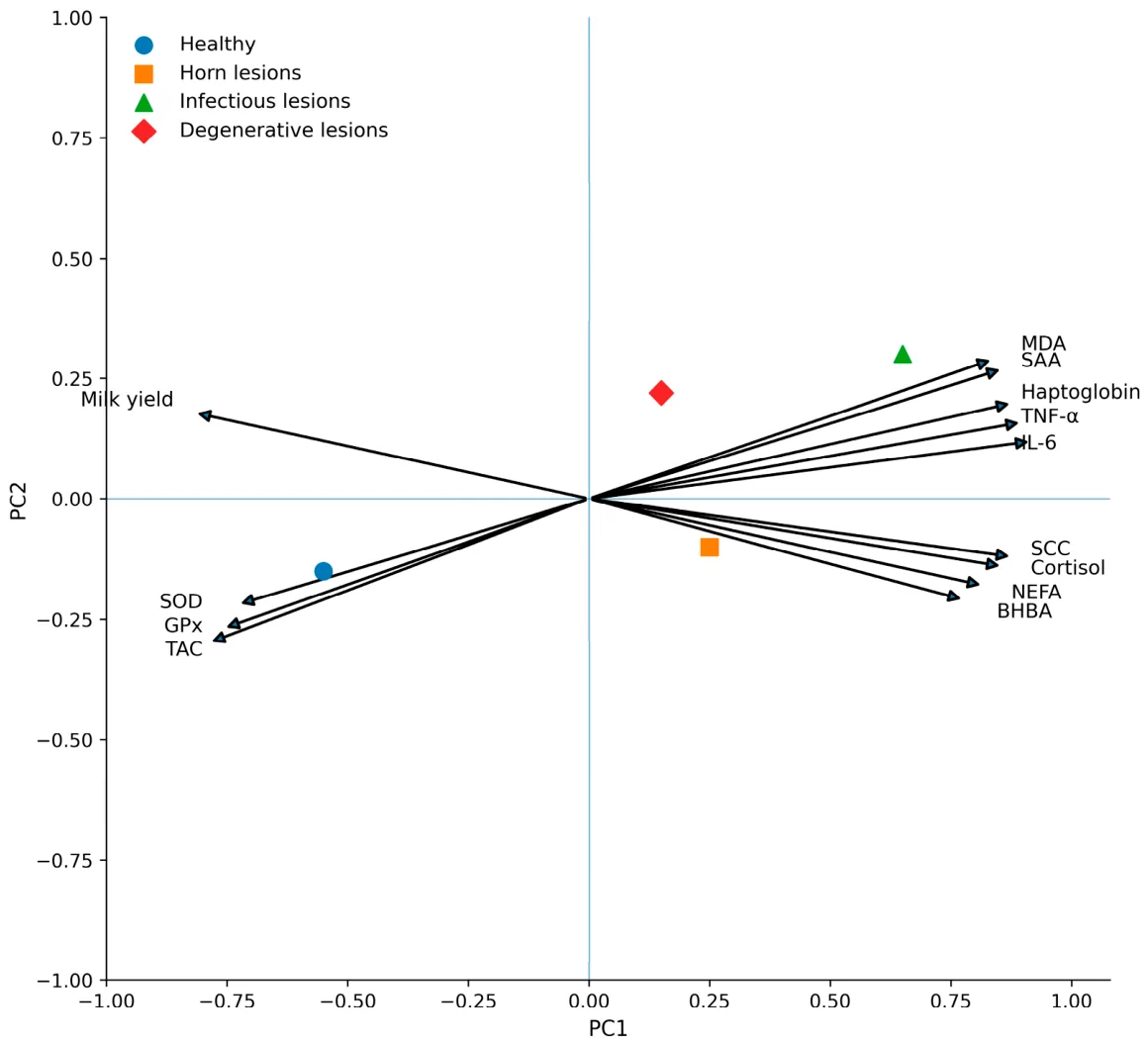
Principal component analysis (PCA) biplot. Arrows represent variable loadings, with their direction and length indicating the direction and magnitude of each variable’s contribution to the principal components.

**Figure 3:**
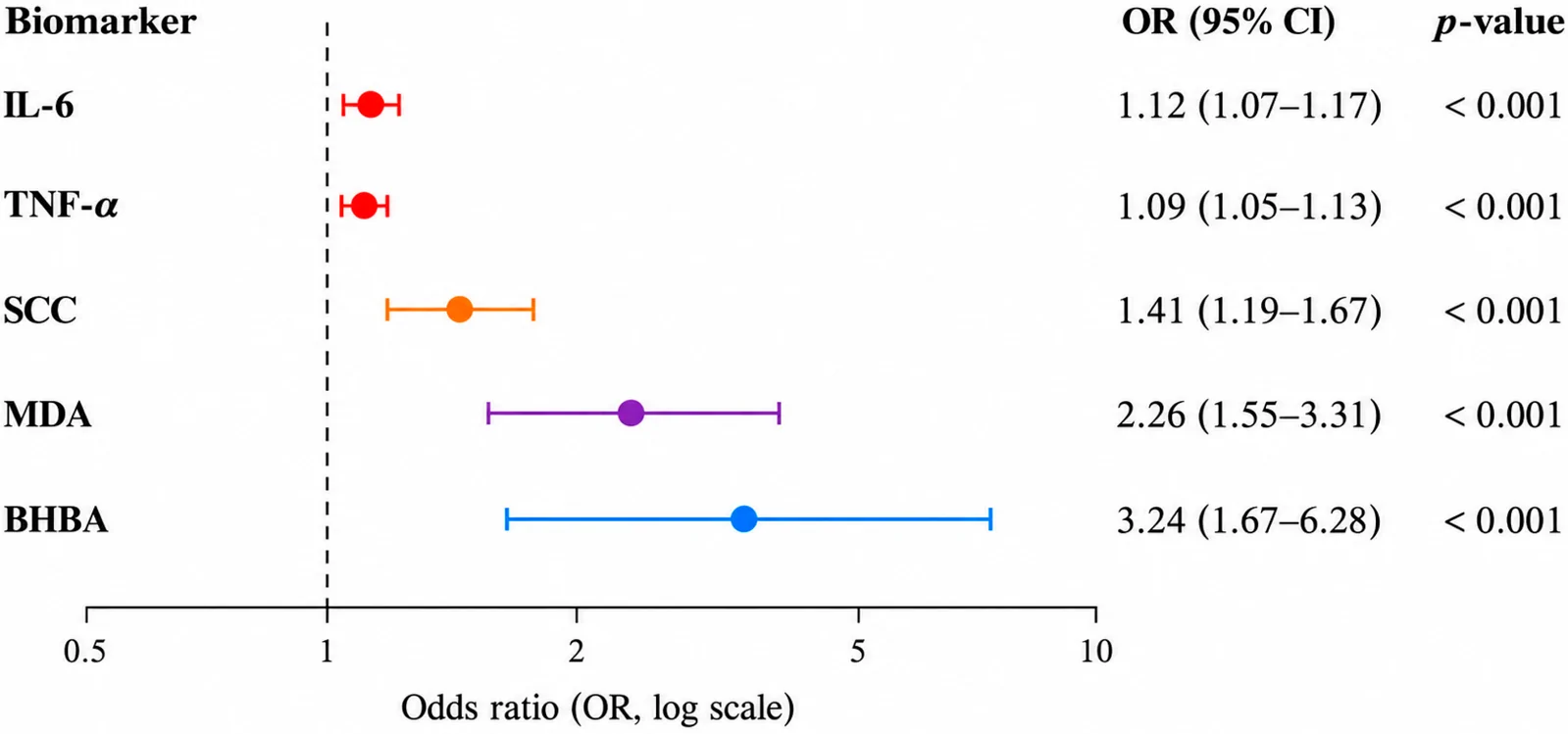
Forest plot showing odds ratios (ORs) with 95% confidence intervals obtained from the multivariate logistic regression model evaluating independent associations between inflammatory, oxidative stress, metabolic, and milk quality biomarkers and lameness in dairy cows. The vertical dashed line indicates the null effect (OR = 1). Biomarkers positioned to the right of the reference line show higher odds of clinically established lameness.

**Table 1:** Inflammatory, oxidative stress, and metabolic biomarkers according to hoof lesion type.

Biomarker	Unit	Healthy Cows (*n* = 210)	Horn Lesions (*n* = 190)	Infectious Lesions (*n* = 32)	Degenerative Lesions (*n* = 13)	*p*-Value
Inflammatory markers						
Haptoglobin	mg/L	18.6 ± 6.4	39.8 ± 10.6	61.2 ± 14.8	31.5 ± 8.9	<0.001
Serum amyloid A (SAA)	mg/L	7.4 ± 2.1	15.2 ± 4.3	24.8 ± 6.1	12.6 ± 3.4	<0.001
IL-1β	pg/mL	18.4 ± 5.2	31.5 ± 7.8	43.2 ± 9.5	25.4 ± 6.3	<0.001
IL-6	pg/mL	11.3 ± 3.7	24.7 ± 6.5	38.5 ± 8.8	19.8 ± 5.6	<0.001
TNF-α	pg/mL	14.7 ± 4.1	27.9 ± 7.3	41.6 ± 9.4	22.8 ± 6.1	<0.001
IL-10	pg/mL	26.8 ± 5.9	21.3 ± 5.2	16.9 ± 4.6	23.4 ± 5.1	<0.001
Oxidative stress markers						
MDA	nmol/mL	2.18 ± 0.46	3.28 ± 0.59	4.17 ± 0.73	2.94 ± 0.52	<0.001
TAC	mmol/L	1.42 ± 0.18	1.18 ± 0.16	0.96 ± 0.15	1.26 ± 0.17	<0.001
GPx	U/mL	72.4 ± 9.5	63.7 ± 8.6	55.4 ± 7.9	66.1 ± 8.8	<0.001
SOD	U/mL	112.8 ± 15.6	99.1 ± 13.8	86.4 ± 12.5	103.7 ± 14.1	<0.001
Catalase	U/mL	38.6 ± 5.9	33.5 ± 5.2	28.6 ± 4.9	35.4 ± 5.3	<0.001
Metabolic markers						
BHBA	mmol/L	0.74 ± 0.18	0.98 ± 0.24	1.36 ± 0.31	0.89 ± 0.22	<0.001
NEFA	mmol/L	0.31 ± 0.09	0.46 ± 0.12	0.68 ± 0.15	0.39 ± 0.10	<0.001
Cortisol	ng/mL	14.6 ± 3.8	20.8 ± 4.6	28.9 ± 5.4	18.7 ± 4.2	<0.001

**Table 2:** Correlation between milk quality traits and inflammatory biomarkers.

Variable	SCC	SCS	IL-6	TNF-α	SAA	MDA	Milk Yield
SCC	1.000	0.94 ***	0.71 ***	0.69 ***	0.73 ***	0.64 ***	−0.48 ***
SCS		1.000	0.68 ***	0.66 ***	0.71 ***	0.60 ***	−0.45 ***
IL-6			1.000	0.81 ***	0.76 ***	0.69 ***	−0.52 ***
TNF-α				1.000	0.72 ***	0.67 ***	−0.49 ***
SAA					1.000	0.71 ***	−0.44 ***
MDA						1.000	−0.39 ***
Milk yield							1.000

*** *p* < 0.001.

**Table 3:** Principal component analysis (PCA) of milk quality, inflammatory, oxidative stress and metabolic biomarkers.

Variable	PC1	PC2	Communality
Milk yield	−0.821	0.184	0.708
Fat	−0.416	0.641	0.584
Protein	−0.587	0.392	0.499
SCC	0.882	−0.115	0.791
SCS	0.861	−0.138	0.760
Haptoglobin	0.873	0.201	0.803
Serum amyloid A	0.854	0.273	0.804
IL-1β	0.846	0.221	0.765
IL-6	0.914	0.118	0.850
TNF-α	0.897	0.163	0.832
MDA	0.841	0.291	0.792
TAC	−0.812	−0.302	0.751
GPx	−0.798	−0.281	0.716
BHBA	0.786	−0.214	0.664
NEFA	0.762	−0.186	0.615
Cortisol	0.808	−0.129	0.670

**Table 4:** Receiver operating characteristic (ROC) analysis of selected biomarkers for discrimination of lame cows.

Biomarker	Cut-Off	AUC (95% CI)	Sensitivity (%)	Specificity (%)	Youden Index	*p*-Value
IL-6	>19.4 pg/mL	0.947 (0.924–0.970)	91.3	88.6	0.799	<0.001
TNF-α	>23.1 pg/mL	0.934 (0.908–0.960)	89.4	85.8	0.752	<0.001
Haptoglobin	>32.4 mg/L	0.921 (0.892–0.950)	86.8	84.2	0.710	<0.001
SAA	>12.8 mg/L	0.913 (0.882–0.944)	84.9	83.1	0.680	<0.001
MDA	>2.94 nmol/mL	0.902 (0.869–0.935)	83.6	81.4	0.650	<0.001
SCC	>268 × 10^3^ cells/mL	0.887 (0.852–0.922)	81.2	79.5	0.607	<0.001
BHBA	>0.94 mmol/L	0.861 (0.822–0.900)	78.4	76.8	0.552	<0.001

**Table 5:** Multivariate logistic regression evaluating independent associations between selected biomarkers and lameness.

Variable	β	SE	Odds Ratio (95% CI)	Wald χ^2^	*p*-Value
IL-6	0.112	0.021	1.12 (1.07–1.17)	28.6	<0.001
TNF-α	0.086	0.019	1.09 (1.05–1.13)	21.9	<0.001
SCC	0.004	0.001	1.41 (1.19–1.67)	18.4	<0.001
MDA	0.814	0.192	2.26 (1.55–3.31)	17.9	<0.001
BHBA	1.176	0.338	3.24 (1.67–6.28)	12.1	<0.001

**Table 6:** Characteristics of the experimental groups.

Parameter	Healthy Cows	Lame Cows
Breed	Polish Holstein-Friesian	Polish Holstein-Friesian
Number of cows	210	241
Hoof health status	Healthy	Hoof disorders

**Table 7:** Association between hoof health status and milk production and milk quality traits.

Parameter	Healthy Cows	Lame Cows	Mean Difference	Cohen’s *d*	*p*-Value
Milk yield (kg/year)	9845 ± 284	8912 ± 437	−933	0.69	<0.001
Fat (%)	4.18 ± 0.34	3.97 ± 0.39	−0.21	0.57	0.002
Protein (%)	3.46 ± 0.18	3.29 ± 0.21	−0.17	0.86	<0.001
Fat-to-protein ratio	1.21 ± 0.10	1.21 ± 0.12	0.00	0.02	0.881
Somatic cell count (×10^3^ cells/mL)	182 ± 79	391 ± 171	+209	1.53	<0.001
Somatic Cell Score (SCS)	2.63 ± 0.71	4.08 ± 1.02	+1.45	1.64	<0.001

**Table 8:** Classification of hoof disorders according to lesion type.

Lesion Category	Hoof Disorder	*n*	%
Horn lesions	Sole ulcer	78	32.4
	Double sole	39	16.2
	Sole hemorrhage	33	13.7
	White line abscess	20	8.3
	Heel ulcer	13	5.4
	Toe ulcer	7	2.9
Infectious lesions	Digital dermatitis	26	10.8
	Interdigital dermatitis	6	2.5
	Interdigital phlegmon	6	2.5
Degenerative lesions	Heel horn erosion	13	5.4
Total		241	100.0

Note: Individual cows could present more than one hoof lesion; therefore, the sum of lesion diagnoses may exceed the number of lame cows. Six lame cows presented combinations of lesions that did not allow their unambiguous assignment to a single lesion category and were therefore included in the overall lame group but excluded from lesion-category comparisons.

## Data Availability

All data generated or analyzed during the study are included within the article.

## Abbreviations

The following abbreviations are used in this manuscript:

Abbreviation	Definition
ANOVA	Analysis of Variance
AST	Aspartate Aminotransferase
AUC	Area under the Curve
BHBA	β-Hydroxybutyrate
CAT	Catalase
CI	Confidence Interval
GPx	Glutathione Peroxidase
GGT	Gamma-Glutamyl Transferase
HSD	Honestly Significant Difference
Hp	Haptoglobin
IL	Interleukin
MDA	Malondialdehyde
NEFA	Non-Esterified Fatty Acids
OR	Odds Ratio
PCA	Principal Component Analysis
ROC	Receiver Operating Characteristic
SAA	Serum Amyloid A
SCC	Somatic Cell Count
SCS	Somatic Cell Score
SD	Standard Deviation
SE	Standard Error
SOD	Superoxide Dismutase
TAC	Total Antioxidant Capacity
TBARS	Thiobarbituric Acid Reactive Substances
TMR	Total Mixed Ration
TNF-α	Tumor Necrosis Factor-Alpha

## References

[1] Alvergnas M., Strabel T., Rzewuska K., Sell-Kubiak E. (2019). Claw disorders in dairy cattle: Effects on production, welfare and farm economics with possible prevention methods. Livest. Sci..

[2] Anagnostopoulos A., Griffiths B.E., Siachos N., Neary J., Smith R.F., Oikonomou G. (2023). Initial validation of an intelligent video surveillance system for automatic detection of dairy cattle lameness. Front. Vet. Sci..

[3] Whay H.R., Shearer J.K. (2017). The impact of lameness on welfare of the dairy cow. Vet. Clin. Food Anim. Pract..

[4] Contreras G.A., Sordillo L.M. (2011). Lipid mobilization and inflammatory responses during the transition period of dairy cows. Comp. Immunol. Microbiol. Infect. Dis..

[5] Sordillo L., Mavangira V. (2014). The nexus between nutrient metabolism, oxidative stress and inflammation in transition cows. Anim. Prod. Sci..

[6] Berry S.L., Read D.H., Famula T.R., Mongini A., Döpfer D. (2012). Long-term observations on the dynamics of bovine digital dermatitis lesions on a California dairy after topical treatment with lincomycin HCl. Vet. J..

[7] Solano L., Barkema H., Pajor E., Mason S., LeBlanc S., Heyerhoff J.Z., Nash C., Haley D., Vasseur E., Pellerin D. (2015). Prevalence of lameness and associated risk factors in Canadian Holstein-Friesian cows housed in freestall barns. J. Dairy Sci..

[8] Solano L., Barkema H., Pickel C., Orsel K. (2017). Effectiveness of a standardized footbath protocol for prevention of digital dermatitis. J. Dairy Sci..

[9] Greenough P. (2007). Kulawizny Bydła.

[10] Green L.E., Hedges V.J., Schukken Y.H., Blowey R.W., Packington A.J. (2002). The Impact of Clinical Lameness on the Milk Yield of Dairy Cows. J. Dairy Sci..

[11] Green L.E., Huxley J.N., Banks C., Green M.J. (2014). Temporal associations between low body condition, lameness and milk yield in a UK dairy herd. Prev. Vet. Med..

[12] Alawneh J.I., Laven R., Stevenson M. (2011). The effect of lameness on the fertility of dairy cattle in a seasonally breeding pasture-based system. J. Dairy Sci..

[13] Archer S.C., Green M.J., Huxley J.N. (2010). Association between milk yield and serial locomotion score assessments in UK dairy cows. J. Dairy Sci..

[14] Mellado M., Saavedra E., Gaytán L., Veliz F., Macías-Cruz U., Avendaño-Reyes L., García E. (2018). The effect of lameness-causing lesions on milk yield and fertility of primiparous Holstein cows in a hot environment. Livest. Sci..

[15] Džermeikaitė K., Krištolaitytė J., Anskienė L., Šertvytytė G., Lembovičiūtė G., Arlauskaitė S., Girdauskaitė A., Rutkauskas A., Baumgartner W., Antanaitis R. (2025). Effects of lameness on milk yield, milk quality indicators, and rumination behaviour in dairy cows. Agriculture.

[16] Cook N.B. (2003). Prevalence of lameness among dairy cattle in Wisconsin as a function of housing type and stall surface. J. Am. Vet. Med. Assoc..

[17] Cook N.B., Nordlund K.V., Oetzel G.R. (2004). Environmental Influences on Claw Horn Lesions Associated with Laminitis and Subacute Ruminal Acidosis in Dairy Cows. J. Dairy Sci..

[18] Plummer P.J., Krull A. (2017). Clinical Perspectives of Digital Dermatitis in Dairy and Beef Cattle. Vet. Clin. N. Am. Food Anim. Pract..

[19] Relun A., Lehébel A., Bruggink M., Bareille N., Guatteo R. (2013). Estimation of the relative impact of treatment and herd management practices on prevention of digital dermatitis in French dairy herds. Prev. Vet. Med..

[20] Abuelo A., Hernández J., Benedito J.L., Castillo C. (2015). The importance of the oxidative status of dairy cattle in the periparturient period: Revisiting antioxidant supplementation. J. Anim. Physiol. Anim. Nutr..

[21] Ayemele A.G., Tilahun M., Lingling S., Elsaadawy S.A., Guo Z., Zhao G., Xu J., Bu D. (2021). Oxidative Stress in Dairy Cows: Insights into the Mechanistic Mode of Actions and Mitigating Strategies. Antioxidants.

[22] Puppel K., Slósarz J., Solarczyk P., Grodkowski G., Kostusiak P., Kalińska A., Balcerak M., Kunowska-Slósarz M., Gołębiewski M. (2024). Assessing the usefulness of interleukin-8 as a biomarker of inflammation and metabolic dysregulation in dairy cows. Int. J. Mol. Sci..

[23] Wnorowska K., Młynek K., Puppel K. (2024). The impact of negative energy balance in holstein-friesian cows on the blood concentrations of interleukin-6 and plasminogen. Metabolites.

[24] Tothova C., Nagy O., Kovac G. (2014). Acute phase proteins and their use in the diagnosis of diseases in ruminants: A review. Veterinární Medicína.

[25] Puppel K., Slósarz J., Grodkowski G., Solarczyk P., Kostusiak P., Kunowska-Slósarz M., Grodkowska K., Zalewska A., Kuczyńska B., Gołębiewski M. (2022). Comparison of enzyme activity in order to describe the metabolic profile of dairy cows during early lactation. Int. J. Mol. Sci..

[26] Solarczyk P., Gołębiewski M., Slósarz J., Puppel K. (2023). Interaction between the Concentration of β-Hydroxybutyric Acid and the Content of Long-Chain Fatty Acids in the Early Stage of Lactation––Comparing Multiparous and Primiparous Cows. Appl. Sci..

[27] Suthar V., Canelas-Raposo J., Deniz A., Heuwieser W. (2013). Prevalence of subclinical ketosis and relationships with postpartum diseases in European dairy cows. J. Dairy Sci..

[28] Pirkkalainen H., Talvio I., Kujala-Wirth M., Soveri T., Orro T. (2022). Acute phase response of sole ulcer, white line disease and digital dermatitis in dairy cows. Vet. Anim. Sci..

[29] Pirkkalainen H., Riihimäki A., Lienemann T., Anttila M., Kujala-Wirth M., Rajala-Schultz P., Simojoki H., Soveri T., Orro T. (2024). Local and systemic inflammation in Finnish dairy cows with digital dermatitis. Animals.

[30] Fritzen A., da Silva A.S. (2025). Inflammation in the Transition Period of Dairy Cows: New Paradigms. Vet. Sci..

[31] Mezzetti M., Bionaz M., Trevisi E. (2020). Interaction between inflammation and metabolism in periparturient dairy cows. J. Anim. Sci..

[32] LeBlanc S. (2010). Monitoring metabolic health of dairy cattle in the transition period. J. Reprod. Dev..

[33] Bobbo T., Penasa M., Cassandro M. (2020). Combining total and differential somatic cell count to better assess the association of udder health status with milk yield, composition and coagulation properties in cattle. Ital. J. Anim. Sci..

[34] Sadiq M.B., Ramanoon S.Z., Mansor R., Mossadeq W.M.S., Syed-Hussain S.S., Yimer N., Kaka U., Ajat M., Abdullah J.F.F. (2024). Potential biomarkers for lameness and claw lesions in dairy cows: A scoping review. J. Dairy Res..

